# Gene Expression Disruptions of Organism versus Organ in Drosophila Species Hybrids

**DOI:** 10.1371/journal.pone.0003009

**Published:** 2008-08-20

**Authors:** Daniel J. Catron, Mohamed A. F. Noor

**Affiliations:** Biology Department, Duke University, Durham, North Carolina, United States of America; Queens University, Canada

## Abstract

Hybrid dysfunctions, such as sterility, may result in part from disruptions in the regulation of gene expression. Studies of hybrids within the *Drosophila simulans* clade have reported genes expressed above or below the expression observed in their parent species, and such misexpression is associated with male sterility in multigenerational backcross hybrids. However, these studies often examined whole bodies rather than testes or had limited replication using less-sensitive but global techniques. Here, we use a new RNA isolation technique to re-examine hybrid gene expression disruptions in both testes and whole bodies from single Drosophila males by real-time quantitative RT-PCR. We find two early-spermatogenesis transcripts are underexpressed in hybrid whole-bodies but not in assays of testes alone, while two late-spermatogenesis transcripts seem to be underexpressed in both whole-bodies and testes alone. Although the number of transcripts surveyed is limited, these results provide some support for a previous hypothesis that the spermatogenesis pathway in these sterile hybrids may be disrupted sometime after the expression of the early meiotic arrest genes.

## Introduction

Hybrid dysfunctions, such as sterility or inviability, result from failed (or novel deleterious) interactions between the genomes of the two parent species. Although conclusive evidence is still lacking, several recent studies have suggested that disruptions in gene expression may be one source for these failed interactions [for reviews,see 1], [2] [but see 3]. For example, disruptions in expression of *Xmrk-2* in some backcross hybrids of *Xiphophorus maculates* and *X. helleri* cause spots on their dorsal fins to spontaneously develop malignant melanomas [4], [5]. Theoretical investigations have also suggested that interspecies hybrids can have low fitness where natural selection has altered interacting molecules, such as the binding affinity between transcription factors and DNA binding sites, independently between the two parent species [6]–[8].

**Figure 1:**
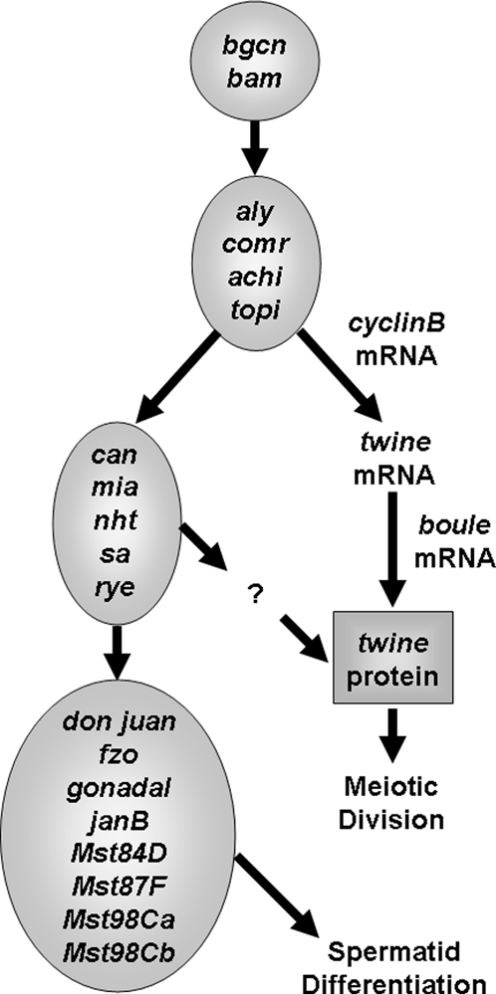
Drosophila spermatogenesis regulatory pathway [adapted from 17], [18].

Disruptions in gene expression have been examined extensively in hybrids of the genus Drosophila [e.g.9], [10], and particularly within the *D. simulans* clade (*D. simulans, D. mauritiana, D. sechellia*). Recent studies have used microarrays to examine disruptions in gene expression in male hybrids of *D. simulans* clade species [11]–[13]. All three studies found many genes severely underexpressed in the hybrids relative to the pure species, and these genes were disproportionately associated with spermatogenesis or other male-specific phenotypes. Michalak and Noor [14] further found that sterility and underexpression of five transcripts were strongly correlated in fifth generation backcross hybrids of *D. simulans* and *D. mauritiana*, and a recent study found that one of these transcripts appears to be directly involved in incompatibilities leading to hybrid sterility [15]. Thus, it is possible that misexpression of male-fertility-essential genes involved in spermatogenesis caused sterility in these hybrids. Underexpressed genes also appear to be more rapidly evolving than genes expressed normally in hybrids [16]. Finally, Moehring et al. [13] overlaid their misexpression results onto part of a known spermatogenesis pathway for *D. melanogaster* [see Figure 1, adapted from 17,18] and found many late-stage downstream loci exhibiting misexpression (e.g., *don juan, gonadal, Mst84D, Mst98Ca, Mst98Cb, Mst87D*), whereas relatively few early-stage loci were misexpressed. This finding may suggest that the spermatogenesis regulatory pathway could be disrupted at a particular stage.

However, some of the studies described above [12], [13] used whole adult bodies for investigating hybrid misexpression, and it is unclear if this approach was misleading. If there were tissue-specific hybrid expression disruptions, then testes-specific misexpression may have been either missed or erroneously inferred (by disruptions in other tissues of male-related transcripts). One approach to address this concern would be to focus expression assays on testes alone, as done by Haerty and Singh [11]. However, Haerty and Singh [11] examined hybrid expression via microarrays designed with probes from a related species (*D. melanogaster*), which has been shown to be less-sensitive [13], [19], and they used both extensive pooling across individuals and an RNA amplification step in their assays. While theirs was a good first approach, a logical next step would be to use biologically replicated quantitative real-time PCRs on cDNA pools derived from testes of single flies to examine the extent of misexpression. Our experience has shown that real-time PCR will sometimes identify expression differences not observed via microarray analyses (e.g., hybrid misexpression of *always early*: [20]).

Here, we present a novel protocol for consistent real-time PCR quantification of transcript abundance from the testes of a single Drosophila. We use this approach on four spermatogenesis-related transcripts to address two questions relating to hybrid misexpression in *Drosophila simulans* clade species, which diverged from each other approximately 250,000 years ago [21]. First, we test whether the same hybrid misexpression in whole bodies is also apparent when testes are examined alone. Second, we test the hypothesis of Moehring et al. [13] that hybrid misexpression is preferentially localized to late-stage downstream loci relative to early-stage loci of known spermatogenetic pathways in this system. If this hypothesis is confirmed, that would further support, albeit not prove, the hypothesis that gene expression disruptions could contribute to hybrid sterility in this system.

## Materials and Methods

### *Drosophila* Strains

*Drosophila simulans* Florida City strain and *D. mauritiana* Synthetic strain were maintained on standard sugar-yeast-agar medium on a 12-hour light-dark cycle at 20°C. Virgin *D. simulans* females were crossed with *D. mauritiana* males. F_1_ males were collected, housed three days post-eclosion, and nucleic acids were extracted immediately thereafter from either testes or whole fly body.

### Development of Custom TaqMan Gene Expression Assays

The *D. melanogaster* DNA sequences of four genes known to be involved in spermatogenesis (*always early* (*aly*), *cookie monster* (*comr*), *don juan* (*dj*), and *Mst84D*) and an endogenous control gene (*Actin5C*), were collected from NCBI (http://www.ncbi.nlm.nih.gov/). The *D. melanogaster* DNA sequence was BLASTed against the *D. simulans* genome using Drosophila Species Genomes BLAST (http://insects.eugenes.org/species/blast/). The *D. simulans* genes were aligned with the orthologous *D. melanogaster* sequences. Primers were designed to sequence regions of the genes with minimal nucleotide differences between the two species. Sequencing was performed for our *D. simulans* Florida City and *D. mauritiana* Synthetic strains. The resulting sequence reads were aligned to reveal polymorphisms between the orthologs. TaqMan probe and primer sets were designed to avoid polymorphisms, while maximizing the efficiency of the amplification. When possible, probes were designed to cross an intron/exon boundary. Probes recognizing the four genes of interest were labeled with a 5′ FAM reporter fluorophore and the endogenous control gene was labeled with a 5′ VIC fluorophore. Probes and primers were synthesized by Applied Biosystems (Foster City, CA). The probe and primer sequences are listed in Table S1.

### RNA Extraction and cDNA Synthesis

Whole-fly RNA extractions were prepared according to a previously published protocol [22], followed by cDNA synthesis using the High-Capacity cDNA Reverse Transcription Kit from Applied Biosystems.

Testes were carefully dissected from anesthetized flies in insect Ringer's solution and immediately processed using the TaqMan Gene Expression Cells-to-C_T_ Kit from Applied Biosystems with a modified protocol (see Methods S1 online for the detailed protocol). We employed both technical and biological replication (3–4 separate testes dissections), with the latter involving independent reverse transcription reactions and subsequent RQ-PCRs from testes of each fly dissected. We also performed both no-template controls and reactions with RNA that was not reverse transcribed (to confirm the absence of genomic DNA contamination). Finally, we did also amplify and sequence both parental strains and F_1_ hybrids to confirm that the F_1_ hybrid samples were heterozygous at all polymorphisms identified (and not contaminants).

### Data Analyses

Real-time quantitative PCR was performed on an ABI 7000 PRISM Sequence Detection System. Raw C_T_ values were collected and analysis was performed according to the 2^−ΔΔ*C*^_T_ method [23], using *Actin5C* to normalize estimates of relative expression. The same general trends were apparent in the raw C_T_ values for the focal genes as in the normalized data, suggesting *Actin5C* normalization did not skew the results. Whole body expression data for *always early* were taken directly from Noor [20]. For visualization in Table 1, relative expression was further normalized on an arbitrary scale where the *D. mauritiana* average was set to 1.00 through simple division.

**Table 1:** Normalized average relative expression of spermatogenesis-related transcripts in whole bodies and testes alone.

Tissue	Transcript	*D. simulans*	*D. mauritiana*	F1 hybrid males
Whole bodies	*always early*	1.210	1.000	0.154
	*cookie monster*	5.086	1.000	0.029
	*don juan*	1.748	1.000	0.371
	*Mst84D*	1.545	1.000	0.116
Testes alone	*always early*	1.011	1.000	0.801
	*cookie monster*	0.602	1.000	0.888
	*don juan*	1.521	1.000	0.610
	*Mst84D*	0.737	1.000	0.310

Statistical analysis was performed with StatView (SAS Institute, Cary, NC). Samples were compared via ANOVA, but qualitatively similar results were obtained when using nonparametric pairwise comparative statistics such as the Mann-Whitney U-test.

## Results

Using our RNA preparations, we were able to obtain real-time PCR quantitations of reverse transcribed genes from both whole bodies and testes alone. Using RNA derived from whole bodies, we found that all four transcripts (*aly, comr, dj, Mst84D*) were all expressed in F_1_ hybrid males at levels significantly lower than in both parent species (see Table 1). One sample of *D. simulans comr* was anomalously higher than the rest (three times higher than the rest), skewing the figure presented; however, excluding this sample still yielded a significant difference.

**Figure 2:**
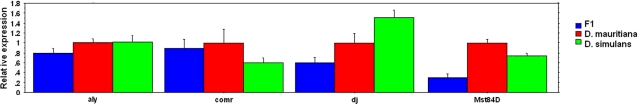
Relative expression of spermatogenesis-related transcripts in testes of *D. mauritiana*, *D. simulans*, and F_1_ hybrid males, bars indicating±1 standard error.

The degree of underexpression was substantially reduced when testes were examined alone (Table 1, Figure 2). For the genes *aly* and *comr*, there was extensive overlap in expression between the F_1_ hybrid samples and both pure-species samples, demonstrating the lack of a consistent difference in expression between hybrids and pure species at these early-acting loci. We did observe a significant difference in expression at *Mst84D* between pure-species and hybrids (F_1_ vs. *D. simulans*, p = 0.0039, F_1_ vs. *D. mauritiana*, p = 0.0007) when testes were examined alone. The difference in testes expression at *dj* was also significant between *D. simulans* and the F_1_ hybrids (p = 0.0022), but one extreme outlier sample of *D. mauritiana* (having expression one third that of all of the other samples) made the expression difference between these samples and the F_1_ not statistically significant. However, if this extreme outlier (which also had much weaker PCR amplification of both the experimental and control genes) was excluded, this difference in expression was also significant (p = 0.0086).

## Discussion

This study presents two conceptual advances and one technical advance for understanding the role of gene expression disruptions in Drosophila hybrid sterility. Studies of hybrid male misexpression (over- or under-expression relative to both pure species) have either focused on whole bodies or on RNA isolated from testes. Here, we use both techniques on hybrids of *Drosophila simulans* and *D. mauritiana*, and we find that some transcripts are underexpressed when the whole body is studied but not underexpressed when testes are surveyed alone. This difference may partially explain the discrepancy in results from the studies of Haerty and Singh (89 underexpressed genes in testes: [11]) and Moehring et al. (502 underexpressed genes in whole bodies: [13]) when surveying hybrids of the same species pairs. Additionally, using assays of four genes, we found some support for the hypothesis of Moehring et al. [13] that genes late in the spermatogenesis pathway (*Mst84D* and *don juan*) are more likely to be underexpressed in hybrid testes than those early in the spermatogenesis pathway (*always early* and *cookie monster*, see Figure 1). While this conclusion is tentative because we cannot necessarily extrapolate from assays of only four loci, it nonetheless suggests the possibility that the spermatogenesis pathway in these sterile hybrids may be disrupted sometime after the expression of the early meiotic arrest genes.

Several hypotheses can explain the difference in detected expression between whole bodies and testes alone. Since the testes appear to be of comparable size and structure in all flies surveyed, some of the transcripts identified by Moehring et al. [13] may be underexpressed in other tissues. However, evidence for non-testes expression of these transcripts in other tissues is minimal. Very low levels of *always early* and *cookie monster* are suggested by microarray analysis of various tissues [24], but RT-PCR or northern blots of gonadectomized or germline-less adult males failed to detect them [18], [25]. Alternatively, there may be developmental (or very broad regulatory) disruptions present in testes of these sterile hybrids that are not apparent with microscopic visualization. For example, if testes of sterile hybrids have reduced testes transcription overall (or even just reduced expression of the control gene), then testes-specific transcripts would be detected as underexpressed in RNA preparations from whole bodies but not necessarily in RNA preparations from testes alone. Consistent with this hypothesis, *donjuan* and *Mst84D* appear to be expressed at more similar levels between testes RNA samples of hybrids and pure species than between whole body RNA samples of hybrids and pure species. Nonetheless, at the present time, the reason for this difference in relative expression remains speculative.

An important aspect of our results is that we do not observe the complete breakdown of this spermatogenesis regulatory pathway in testes of sterile hybrids. Instead, multiple transcripts are expressed at levels within the range of that observed within species. Relatedly, in sterile F_1_ hybrids of these species (and various introgression lines), spermatogenesis appears to proceed to and through meiosis and arrest thereafter [26], [27], but this conclusion relies on microscopic observations that may have missed subtle defects. If disruptions in particular segments of this transcriptional network (Figure 1) are associated with hybrid sterility, it may be possible to use a directed “candidate gene” approach to identify specific failed interactions. If we can extrapolate from the results presented here, a logical place to look for such failed interactions would be with interactions involving late-acting meiotic-arrest genes of the *cannonball* (*can*) class [28].

With help from Applied Biosystems, we have developed a protocol for consistent real-time PCR quantification of transcript abundance from the testes derived from single Drosophila. While expression analyses from even single cells have been possible for some time [e.g., 29], the preparations sometimes rely upon the use of cultured cells, polyadenylated transcripts, RNA amplification steps [e.g., 30], or other manipulations or limitations. Prior to using this approach, we attempted RNA isolation and amplification using a commercially available kit, but the amplified RNA degraded after a few freeze-thaws. Other more direct RNA isolation approaches we attempted did not have high repeatability across technical replicates or had issues including false amplification or contamination. The approach utilized here involves RNA isolation from testes followed by cDNA synthesis for use as a template in RQ-PCR. Replicates of our genomic DNA control samples typically exhibited amplification cycle thresholds of within 0.2 C_T_. Overall, this approach provides an accurate and robust method for the confirmation of tissue-specific expression variation at specific loci identified by a microarray experiment or other high-throughput system.

Recent work has begun to expand the range of species examined for hybrid gene expression disruptions. For example, research on Xenopus and Mus hybrids also identified large panels of male- or testis-specific transcripts misexpressed [31], [32] but intriguingly, Xenopus hybrid testes are also microRNA depleted [33]. With the growing availability of whole-genome sequence assemblies and novel methods for using deep sequencing/tag profiling to assess expression of a wide range of transcripts (including small regulatory RNAs), we should soon be able to assess generalities associated with expression, and misexpression, in sterile hybrids across broader taxonomic groups.

## Supporting Information

Table S1 Probes/primers. TaqMan Assay components designed to equally detect aly, comr, donjuan, or Mst84D gene expression in both our Drosophila simulans Florida City and D. mauritiana Synthetic strains.
(0.04 MB DOC)

Methods S1 Detailed RNA isolation & RT-PCR protocols.
(0.08 MB DOC)
