# Supplementary material for: Gene Expression Disruptions of Organism versus Organ in Drosophila Species Hybrids
Source: PLoS One. 2008 Aug 20;3(8):e3009. doi: 10.1371/journal.pone.0003009 (PMC2500191; doi:10.1371/journal.pone.0003009)
Supplement: Table S1 — Probes/primers. TaqMan Assay components designed to equally detect aly, comr, donjuan, or Mst84D gene expression in both our Drosophila simulans Florida City and D. mauritiana Synthetic strains. (0.04 MB DOC) [file pone.0003009.s001.doc]

Supplementary Table 1 – TaqMan Assay components designed to equally detect *aly, comr, donjuan,* or *Mst84D* gene expression in both our *Drosophila simulans* Florida City and *D. mauritiana* Synthetic strains.

Comr

| Component | Sequence | Mods |
| --- | --- | --- |
| Fd Primer | GCGGGTGTTCCATATTTCATTGTC |  |
| Rv Primer | TTGAAGGATGTGCGTAAATCCACA |  |
| Probe | TTGGCCCACGGATTC | FAM/NFQ |

Donjuan

| Entity | Sequence | Mods |
| --- | --- | --- |
| Fd Primer | GCCTCACCACATCAATGTCCTT |  |
| Rv Primer | TCCAAATCAGGGGCAAACAAAATTT |  |
| Probe | AACTTTAAGGAAGCGATGA | FAM/NFQ |

Mst84D

| Entity | Sequence | Mods |
| --- | --- | --- |
| Fd Primer | GCCAAATGGTCCGCAACAAG |  |
| Rv Primer | AAGGTCCACAGCAGGGAC |  |
| Probe | CCGCACGGACCACAT | FAM/NFQ |

Aly

| Entity | Sequence | Mods |
| --- | --- | --- |
| Fd Primer | GCAGGATCCCTACTCCAATGATTTT |  |
| Rv Primer | CAAGAGATTGTACAACTTCC |  |
| Fd Probe | CGGCTTGTCCAGGAAGGA | FAM/NFQ |

Actin 5C

| Entity | Sequence | Mods |
| --- | --- | --- |
| Fd Primer | GGCACCCGAGGAGCAC |  |
| Rv Primer | AGATGACCCAGATCATGTTCGAGA |  |
| Probe | CCCGTGCTGCTGACCG | VIC/NFQ |
