# Supplementary material for: Gene Expression Disruptions of Organism versus Organ in Drosophila Species Hybrids
Source: PLoS One. 2008 Aug 20;3(8):e3009. doi: 10.1371/journal.pone.0003009 (PMC2500191; doi:10.1371/journal.pone.0003009)
Supplement: Methods S1 — Detailed RNA isolation & RT-PCR protocols. (0.08 MB DOC) [file pone.0003009.s002.doc]

Reagents and Consumables Used in Protocol

| Item | Source | Catalog No. | Quantity |
| --- | --- | --- | --- |
| TaqMan Gene Expression Cells-to-CT Kit | Applied Biosystems | AM1728 | 100 Reactions |
| 0.5 mL Pellet Pestle, Blue | Kontes | 749521-0590 | 100/pk |
| 0.5 mL RNase-Free Pestle Tube | Kontes | 749510-0590 | 100/pk |
| MicroAmp Optical 96-well Plates | Applied Biosystems | N801-0560 | 10/bx |
| Microseal ‘B’ Film | Bio-Rad | MSB-1001 | 100/pk |
| DEPC Water, Nuclease Free | EMD | 9610 | 1 L |
| RNase Away | VWR | 72830-022 | 250 mL |
| DNA Away | VWR | 53509-506 | 250 mL |

Collection

1. Collect at least three different flies from each line to be tested; it is better to have twice this to account for mistakes, waste, and outliers
2. Flies should be processed on the third day post-eclosion

Dissection

1. Prepare the Lysis + DNase I Solution and chill on ice prior to dissection:
   1. Dilute 0.5 µL DNase I in 49.5 µL Lysis Solution per reaction
   2. Add 20 µL Lysis + DNase I Solution to each 0.5 mL RNase-free microcentrifuge tube
2. Briefly expose flies to CO_2_ to anesthetize them
3. Place a clean microscope slide on the stage of the dissecting microscope and add two small droplets of insect Ringer’s solution on the slide
4. Place a single fly within a few millimeters of one of the droplets of solution
5. Turn the fly so that the head is facing left
6. Gently gasp the head/thorax with a pair of forceps in the left hand and gently tear the abdomen open with a pair of needle-nosed forceps in the right hand
7. Spread the contents of the abdomen into the nearest droplet of Ringer’s solution
8. Identify the testes as bright yellow with a spiral-shape
9. Tease the testes away from the fascia and other tissue, while taking great care to keep the testes walls intact
10. Should the testes become punctured, Drosophila sperm is easy to identify as long white wisps that will quickly spread through the solution; if this happens, discard the dissection and begin again
11. Transfer one pair of testes from each fly to a single 0.5 mL microcentrifuge tube containing Lysis + DNase I Solution
12. Continue dissecting testes for the number of biological replicates required for the experiment

Lysis

1. Homogenize the testes thoroughly with a pestle that securely fits the mortar
2. Collect debris from the pestle with remaining 30 µL Lysis + DNase I Solution
3. Incubate homogenized testes at room temperature for 15 minutes
4. During incubation, prepare the following:
   1. Thaw Stop Solution, flick tube to mix **(do not vortex)**, place on ice
   2. Place 20x RT Enzyme Mix on ice
   3. Place 2x RT Buffer on ice
5. Pipet 5 µL Stop Solution directly into each lysis reaction and mix by pipetting up and down 5 times
6. Incubate at room temperature for 2 minutes
   (can be stored at -80°C for up to two months)

Reverse Transcription

1. Reserve and program a thermal cycler for the RT reaction
   1. Step 1 (reverse transcription): 37°C for 60 minutes
   2. Step 2 (RT inactivation): 95°C for 5 minutes
   3. Step 3 (hold): 4°C *ad infinitum*
2. Prepare RT Master Mixes according to the table below:

RT+ Master Mix

| Component | 1x (µL) |  |  |
| --- | --- | --- | --- |
| 2x RT Buffer | 25.0 |  |  |
| 20x RT Enzyme Mix | 2.5 |  |  |
| Nuclease-free water | 12.5 |  |  |
|  | 40.0 |  |  |

RT– Master Mix (Non Amplification Control, NAC)

| Component | 1x (µL) |  |  |
| --- | --- | --- | --- |
| 2x RT Buffer | 25.0 |  |  |
| 20x RT Enzyme Mix | 0.0 |  |  |
| Nuclease-free water | 15.0 |  |  |
|  | 40.0 |  |  |

1. Add 10 µL sample lysate and 40 µL RT Master Mix to PCR tubes
2. Include a Genomic Template Control (GTC) and a Non-Template Control (NTC, use water instead of template in RT+ reaction)
3. Mix reactions gently and briefly spin down
4. Run reverse transcription reaction

RQ-PCR

1. Place ABI TaqMan Gene Expression Master Mix on ice
2. Prepare standard curve series (1x, 0.5x, 0.1x, 0.05x, 0.01x), diluted in water
3. Reserve and program the ABI Prism 7000 SDS for the RQ-PCR reaction
   1. Step 1 (UNG incubation): 50°C for 2 minutes
   2. Step 2 (enzyme activation): 95°C for 10 minutes
   3. Step 3 (PCR): 95°C for 15 seconds; 55°C for 1 minute (repeat 40 times)
   4. Select dissociation curve protocol
4. Prepare the RQ-PCR Master Mixes in triplicate for each sample;
   controls must include GTC, NTC, and Non-Amplification Control (NAC, water as template is RQ-PCR) samples

| Component | 1x (µL) |  |  |
| --- | --- | --- | --- |
| 2x TaqMan Master Mix | 10.0 |  |  |
| 20x Assay Mix (GOI) | 1.0 |  |  |
| 20x Assay Mix (EN) | 1.0 |  |  |
| cDNA | 2.0 |  |  |
| Nuclease-free water | 6.0 |  |  |
|  | 20.0 |  |  |

GOI = Gene of Interest; EN = Endogenous Normalizer (e.g., Actin 5C)

1. Program RQ-PCR in thermal cycler and run samples

Analysis

1. Set appropriate threshold and baseline parameters
2. Export the data and import data into spreadsheet
3. Group the data according to the detector, with matching endogenous normalizer
4. Observe the C_T_ values for all controls and note any inconsistencies or unexpected results.
5. The controls shall no longer be used in calculations
6. Create a linear regression from the standard curve samples, with the concentration as the *x* (or dependent) variable and the Ct value as the *y* (or independent) variable
7. Calculate the correlation coefficient (R^2^ value) and best-fit line from the data
8. If the correlation coefficient value is not greater than 0.95, the data set is suspect and should be discarded
9. The slope from the best-fit line should be close to -3.3219, which would yield a 100% efficiency
10. Calculate the efficiency, but plugging your slope into the following equation:
11. If your efficiency is too far off of 100% (say 90% ≥ efficiency ≥ 110%), any variance is error and the data should be suspect
12. Create four new columns in the spreadsheet with the following headings: ∆Ct, ∆∆Ct, Fold, and Relative
13. For the ∆Ct column, subtract the unknown Ct value from the endogenous normalizer Ct value
14. For the ∆∆Ct column, take the difference of the first unknown’s ∆Ct from every subsequent ∆Ct, such that the first unknown’s cell will have a value of 0
15. For the Fold column, use the following formula “=1/(2^(∆∆Ct))” is the reciprocal of the amplification of the ratio between unknown and endogenous normalizer
16. Finally, for the relative column, divide the Fold for the first unknown by every other Fold, such that the first unknown will have a value of 1, with all other being relative to it
